# X-ray diffraction reveals the intrinsic difference in the physical properties of membrane and soluble proteins

**DOI:** 10.1038/s41598-017-17216-1

**Published:** 2017-12-05

**Authors:** Xavier Robert, Josiane Kassis-Sahyoun, Nicoletta Ceres, Juliette Martin, Michael R. Sawaya, Randy J. Read, Patrice Gouet, Pierre Falson, Vincent Chaptal

**Affiliations:** 1 Molecular Microbiology and Structural Biochemistry institute, UMR5086 CNRS Univ-Lyon, F-69367 Cedex 7, Lyon France; 2 Molecular Biology Institute, Department of Chemistry and Biochemistry, University of California, Los Angeles, CA 90095 USA; 3 Howard Hughes Medical Institute, University of California, Los Angeles, CA 90095 USA; 4 Department of Haematology, Cambridge Institute for Medical Research, University of Cambridge, Wellcome Trust/MRC Building, Hills Road, Cambridge, CB2 0XY England

## Abstract

Membrane proteins are distinguished from soluble proteins by their insertion into biological membranes. This insertion is achieved via a noticeable arrangement of hydrophobic amino acids that are exposed at the surface of the protein, and renders the interaction with the aliphatic tails of lipids more energetically favorable. This important difference between these two categories of proteins is the source of the need for a specific handling of membrane proteins, which transpired in the creation of new tools for their recombinant expression, purification and even crystallization. Following this line, we show here that crystals of membrane proteins display systematically higher diffraction anisotropy than those of soluble proteins. This phenomenon dramatically hampers structure solution and refinement, and has a strong impact on the quality of electron-density maps. A farther search for origins of this phenomenon showed that the type of crystallization, and thus the crystal packing, has no impact on anisotropy, nor does the nature or function of the membrane protein. Membrane proteins fully embedded within the membrane display equal anisotropy compared to the ones with extra membranous domains or fusions with soluble proteins. Overall, these results overturn common beliefs and call for a specific handling of their diffraction data.

## Introduction

Membrane proteins are the link between the cell and its environment, being the means of exchange through a lipid bilayer that is otherwise impermeable. They are for example involved in signal transduction, solute exchange, energy production or protection of the cell from toxic agents, and are therefore essential components to life. The insertion of membrane proteins into biological membranes requires them to have a special arrangement of amino acids, with the membranous part having hydrophobic residues pointing towards the lipids, while the parts accessible to the solvent display a more similar amino acid arrangement to soluble proteins. It is this membranous region that has created hurdles to their studies, requiring the development of specific tools for their handling, from the production of recombinant proteins to their crystallization^1^.

Once these hurdles have been overcome and diffracting quality crystals have been achieved, it is very often referred in the membrane proteins community to strong anisotropic diffraction, and regularly combined with low resolution, which dramatically complicates structure solving and analysis. The use of the UCLA Diffraction Anisotropy Server (https://services.mbi.ucla.edu/anisoscale/)^2^ to perform ellipsoid truncation and map sharpening is widely spread, with the typical image of intensity falloff along the a*, b*, c* reciprocal cell directions depicted next to the table of crystallographic statistics. Even recently, two major membrane protein structures required experts in the field of crystallography for their structure solution, with extensive explanation in the material and methods section on how they dealt with their strong anisotropy^3,4^. In a few extreme cases, the intensity falloff between the strong and weak primary directions of the crystal was so marked that data-reduction software could not process correctly the datasets to include the high resolution shells^5,6^. In order to overcome this barrier, ellipsoidal truncation was performed on the images before standard data reduction and data phasing procedures.

Diffraction anisotropy is defined from a 2003 study from Popov & Bourenkov who reported the diffraction of 72 macromolecule crystals made of proteins of different molecular weights and folds, and belonging to distinct space groups, that diffracted X-rays to 0.9 Å resolution^7^. They showed that the observed reflections follow a typical intensity falloff as a function of resolution. Diffraction anisotropy is defined as a deviation from this experimental curve, where intensity falls off differently according to directions in reciprocal space^8^. In that respect, the pattern of diffraction intensities is not homogeneously distributed in spherical shells depending on scattering angles and can be described as ellipsoids^2,9^. These experimental X-rays observations are generally linked to defects in crystal packing and/or anisotropic vibrations in the crystallized macromolecules. The impact of such a phenomenon on crystallographic refinement can be dramatic in the case of severe anisotropy, leading to lack of details in final electron density maps. This in turn leads to many problems in model building with a lack of confidence on the position of amino acids and finally a poor structural model.

The current belief is that diffraction anisotropy is originating from fewer crystal contacts within the lattice in specific directions of space, and have been reported as such for a few cases of soluble and membrane proteins^2,10–13^. Despite these observations, we were also faced with many cases where crystals diffracted very anisotropically but without any obvious absence of contacts in the crystal packing, which goes against the above assumption.

To clarify and investigate further this phenomenon, we conducted a systematic study of diffraction anisotropy by using experimental structure factors available at the protein data bank (PDB). We focused especially on membrane protein crystals, benefitting from their significant number to conduct meaningful statistics. We show that membrane proteins behave differently than soluble proteins towards X-ray diffraction, and confirmed that they display more anisotropy. We investigated the distribution of anisotropy over resolution that allows investigators to compare their degree of anisotropy to all available entries. We showed that the lack of crystal contacts is generally associated with higher anisotropy but not always. Elaborating further on this fact, we showed that there is no statistical difference among classes of membrane proteins (Channels *vs* receptors *vs* transporters, etc.) and that the type of crystallization (in-detergent *vs in-meso*) has no influence on the anisotropy either despite the increasing number of crystal contacts observed *in-meso*. We showed that the presence of extra-membranous domains does not influence the anisotropic property of the crystal. Our statistical results reveal the fact that the membrane protein itself influences diffraction anisotropy.

## Results

### Membrane proteins diffract X-rays more anisotropically than soluble proteins, and the two types of proteins do not behave similarly towards X-ray diffraction

**Figure 1:**
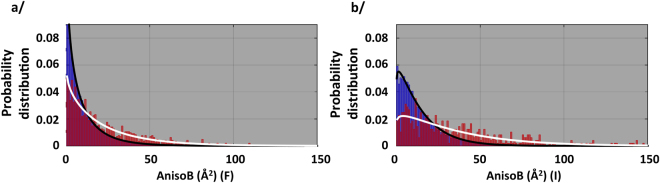
Probability distribution fonction of anisotropy. Soluble proteins and membrane proteins are depicted as blue and red bars respectively. (**a**) Anisotropy calculated on amplitudes (F). (**b**) Anisotropy calculated on intensities (I). The distribution fits (Weibull distributions) are shown as black line for soluble proteins and white line for membrane proteins.

In order to have a broad view of diffraction anisotropy and to grasp this phenomenon on a large scale, we downloaded the whole PDB and calculated anisotropic delta-B values for each entry. We separated membrane proteins from soluble proteins from this database for comparison. We first calculated delta-B values on structure factors processed as amplitudes, for historical reasons and since it represents the most populated format available granting better statistics. Figure 1a displays the probability distribution function of anisotropy. For soluble proteins (blue bars, representing more than 75,000 PDB entries), the distribution fits remarkably with a Weibull distribution (black line), which suggests that all these structures obey the same set of physical rules. Contrary to this behavior, the membrane proteins distribution (red bars representing 1414 PDB entries, white line) is distinct from the soluble proteins one, also best fitted by a Weibull distribution but with different shape and scale parameters. This observed difference in distribution is highly statistically significant (Fig. S1a). This is strong evidence that the two types of proteins do not have the same range of physical parameters describing their motions and disorder, as revealed by X-ray diffraction, and thus indicates that they have distinctive characteristics.

Membrane proteins display an average anisotropy of 23.5 Å^2^ compared to 10.7 Å^2^ for soluble proteins (Table S1). Note that the amplitudes deposited for some structure factor files in the PDB were corrected for anisotropy. We cannot distinguish these files from unmodified ones, and they would tend to have a low anisotropy, which would distort the analysis. However, this practice would if anything tend to mask the differences we have observed between membrane and soluble proteins.

To overcome this problem, we performed the same analysis with delta-B values calculated on intensities using an intensity-based likelihood target that yields more reliable estimates of anisotropy^14^. The results (Figs 1b and S1b) reveal the same difference in distribution and statistical relevance. Note that the distributions are slightly shifted towards higher anisotropy. This can be explained by the fact that application of the French & Wilson algorithm to produce amplitudes tends to inflate the weaker reflections. Calculated this way, membrane proteins display an average anisotropy of 34.4 Å^2^ compared to 15.7 Å^2^ for soluble proteins (Table S1).

Overall, these results confirm previous postulates and accepted views that membrane proteins diffract X-rays more anisotropically, but quite strikingly reveal the fact that they do not behave the same way towards X-ray diffraction compared to soluble proteins.

Since the number of PDB entries with deposited intensities is much smaller for membrane proteins, the break down of membrane proteins into categories described below would result in too few entries in each category to allow for significant statistics. The rest of the analysis will thus be conducted on anisotropy values calculated on amplitudes, vouched by the fact that the calculation on intensities yields the same significance.

### Diffraction anisotropy is not strictly dependent on the total number of crystal contacts

It is widely accepted that diffraction anisotropy from macromolecular crystals is related to a spatial dependence of crystal contacts and/or to solvent content. To investigate this, on a first approach we evaluated the anisotropy as a function of total number of crystal contacts to grasp general trends. The total number of crystal contacts measured between the asymmetric unit and the symmetry-generated molecules was divided by the number of atoms present in the asymmetric unit in order to rationalize the impact of crystal contacts on a structure (calculated including ligands such as lipids in the contacts) (Fig. S2a). The graph shows a trend where the anisotropy varies inversely with the number of crystal contacts, which supports the idea introduced above. Surprisingly though, there is a large variation in anisotropy for any given number of crystal contacts, and there are substantial numbers of structures that do not obey the overall trend. For instance, crystals with few contacts can have observed anisotropy ranging from 0 to the highest cutoff of 150 Å^2^. The number of crystal contacts is therefore not the sole factor influencing diffraction and no direct correlation can be drawn. Membrane protein crystals on average have a lower crystal contacts ratio, 0.08, compared to 0.19 for soluble proteins (Fig. S2b, Table S1). We have not directly investigated crystal contact directionality, as this requires the development of specialized algorithms for large scale studies and will be examined in a follow-up study. Nonetheless, we will provide evidence of directionality for membrane proteins below, *via* the analysis of types of crystallization (detergent *vs* LCP) and *via* the analysis of the presence of extra-membranous domains compared to proteins fully embedded in the membrane.

In addition, anisotropy is not directly correlated with solvent content (Fig. S3). Membrane proteins have higher solvent content with an average of 63.11% compared to soluble proteins (51.11%) (Table S1).

### Relationship between anisotropy and resolution

**Figure 2:**
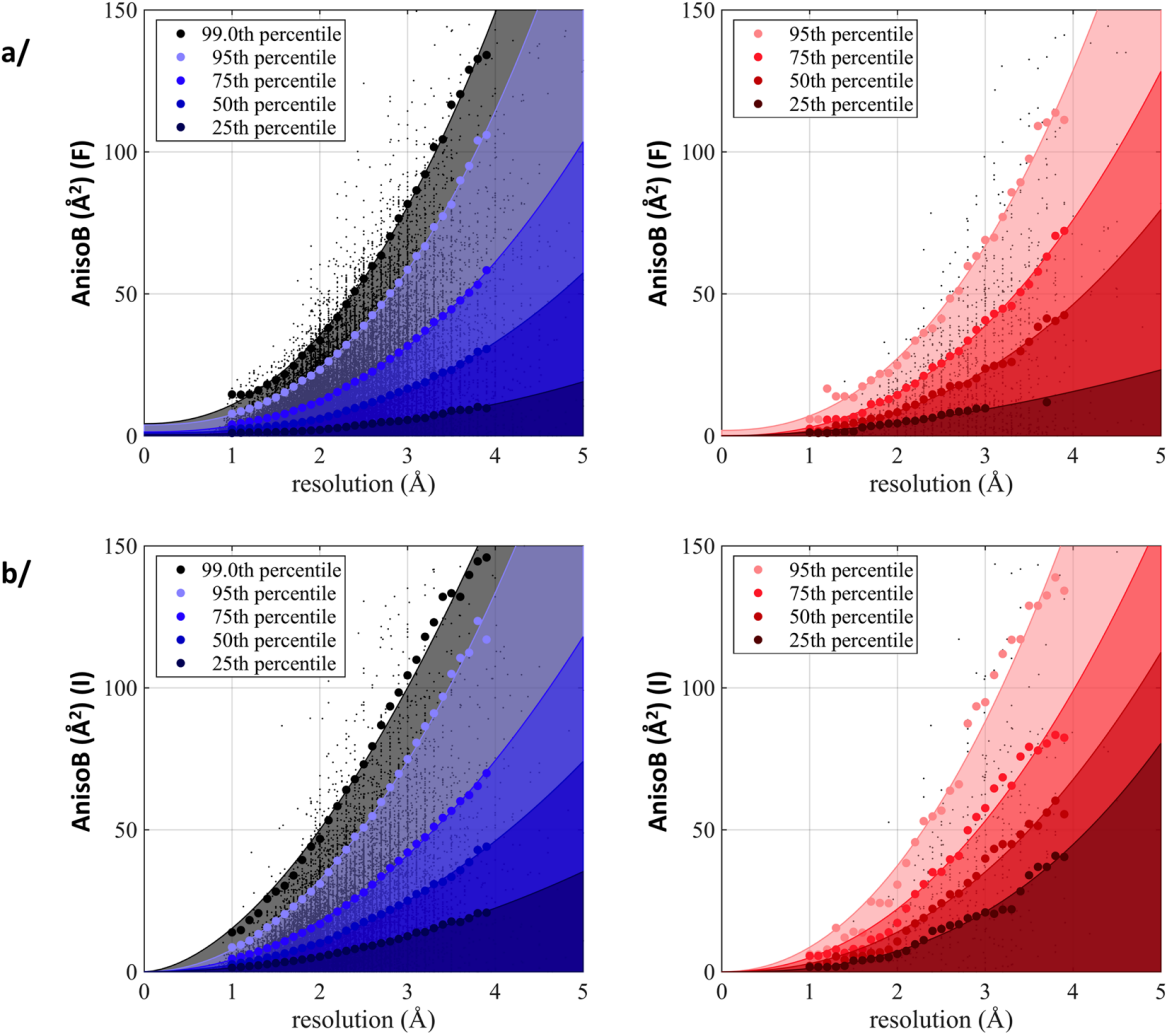
Anisotropy as a function of resolution. Values of anisotropy were calculated on amplitudes (**a**) or intensities (**b**). Soluble proteins are depicted in blue, membrane proteins in red. Each dot represents an entry (soluble proteins: 74,928 amplitudes and 22,985 intensities data; membrane proteins: 1,414 amplitudes and 489 intensities data). The bold dots represent the 25^th^, 50^th^, 75^th^ or 95^th^ percentile per resolution bin, fitted with a power law. For soluble proteins, the 99^th^ percentile is also represented. The area under the fit has been filled with transparent colors according to the percentile.

It is interesting to note that anisotropy increases as the resolution of the dataset worsens (Figs 2a, S4). A range of anisotropy values can be observed for a given resolution, from 0 Å^2^ to an apparent threshold value dependent on resolution. We identified the 25^th^, 50^th^, 75^th^ and 95^th^ percentile of anisotropy values in each resolution bin, which allowed to remarkably fit the data with a power-law model. This representation allows for investigators to compare their datasets to all available entries and to better grasp the degree of diffraction anisotropy. It should also be noted that resolution is a user-defined parameters, and criteria are both user-dependent and evolving with time. Furthermore, in the case of anisotropic data, some users cut their data at the highest resolution of the best diffraction direction, and some others cut their data when the lowest diffracting direction reaches a given threshold value (for example: F/σ(F) of 2). This explains why at lower resolution, when anisotropy is also the strongest, the observed spread in anisotropy is magnified. The percentile-fit reveals that membrane proteins display higher anisotropy than soluble proteins for each resolution bin (Fig. S5a), exemplified by the fact that the 95^th^ percentile of membrane proteins corresponds to the 97.3^th^ percentile of soluble proteins. This result brings to light the intrinsic contribution of the nature of the protein to the phenomenon of X-ray diffraction. Membrane proteins diffract to lower resolution than soluble proteins, on average 2.76 Å compared to 2.13 Å for soluble proteins. (Fig. S4, Table S1). The same behavior is observed when anisotropy is calculated on intensities (Figs 2b, S5b).

### Membrane proteins crystallized in detergent or in LCP display similar anisotropy

Since membrane proteins are surrounded by detergents, the question naturally arises whether crystallization in Lipidic Cubic Phase (LCP) could change the anisotropy of X-ray diffraction to be more like that of soluble proteins? Indeed, detergents form a bulky corona around the membranous region of the membrane protein^15,16^ and are very mobile. These properties would result in fewer crystal contacts in the direction of the detergent corona. Crystallization in LCP is thought to alleviate this problem by increasing the amount of crystal contacts notably *via* lipids, and also displaying a different type of crystallization (Type I *vs* Type II in Detergents) (Fig. S5c).

**Figure 3:**
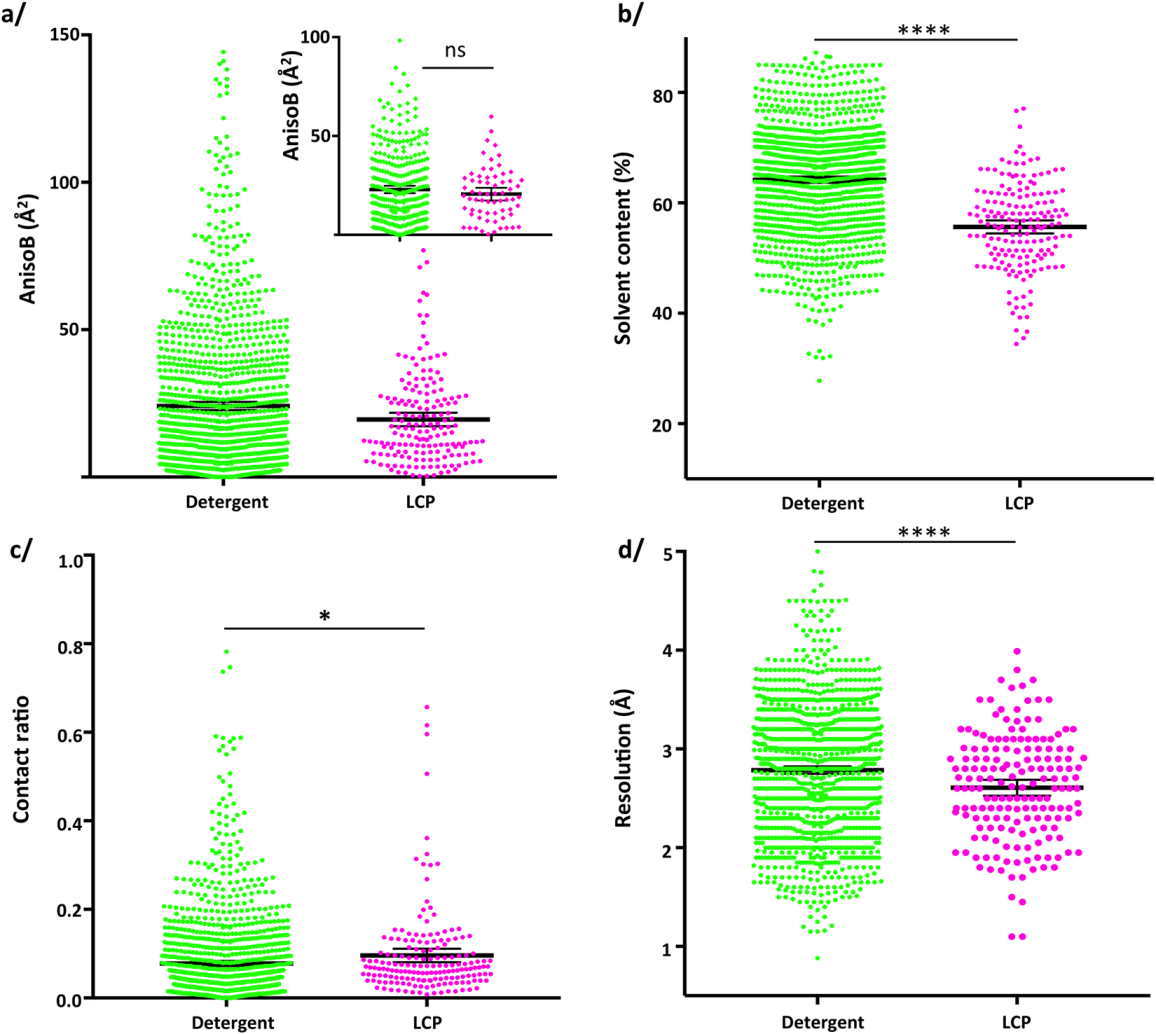
Comparaison of membrane proteins crystallized in detergents *vs* LCP. (**a**) AnisoB: T-test two-tailed p-value = 0.0009; M-W two-tailed p-value = 0.9733. Inset: comparison of anisotropy for structures between 2.5 and 3 Å resolution. Student’s T-test with Welch correction: two-tailed p-value = 0.22; M-W two-tailed p-value = 0.85. (**b**) Solvent Content: T-test two-tailed p-value < 0.0001; M-W two-tailed p-value < 0.0001. (**c**) Crystal contacts ratio: T-test two-tailed p-value = 0.0274; M-W two-tailed p-value < 0.0001. (**d**) Resolution: T-test two-tailed p-value < 0.0001; M-W two-tailed p-value = 0.0007.

Membrane proteins crystallized in detergents have higher solvent content (64.1%) than the ones crystallized in LCP (55.6%) (Fig. 3b, Table S1), have a somewhat lower crystal contacts ratio (0.078 *vs* 0.096) (Fig. 3c) due to the contacts brought by lipids in LCP, and diffract to lower resolution (2.79 Å *vs* 2.61 Å) (Fig. 3d). Yet, the difference in anisotropy is very low, and probably inconsequential (Fig. 3a). On one hand, there is undoubtedly a strong statistical significance that a difference between the two population means exists (using parametric statistics), but the difference will only be between 2 and 7 Å^2^, which is a very small distinction in anisotropy with negligible influence on the quality of the final electron-density maps. On the other hand, using non-parametric statistics to investigate differences in medians, these two categories of proteins behave exactly the same. It should be noted that it is extremely rare that these two tests give rise to opposed significance, and argue in the sense that the statistical significance observed on the means is definitely very small or raises concerns about the true significance observed.

To clarify this inconsistency on the statistical significances, the comparison was performed on structures solved between 2.5 and 3 Å resolution, in order to avoid bias introduced by the higher resolution of LCP crystals (Fig. 3a, inset). It clearly shows that there is no difference between these two types of membrane protein crystallization.

Finally, membrane protein structures crystallized both in detergent and LCP were compared. Table S2 shows the anisotropy for each entry, revealing the complete overlap of the values.

### No difference in anisotropy between different classes of membrane proteins

Since there is limited influence of the crystallization method on diffraction anisotropy, could the nature of the proteins influence anisotropy? Membrane proteins are classically divided by their type of inclusion in the membrane. Three types are observed; two are polytopic (exposed to both sides of the membrane) with the trans-membrane segment being all-alpha-helical or all-beta-sheet, and one defined as monotopic (exposed to only one side of the membrane) and being anchored to the membrane with hydrophobic loops, helices or modified lipids (Fig. S5d).

No statistical difference is observed between the types of insertion into the membrane (Fig. S6a, Table S1), indicating that the class of membrane proteins has no influence on the distribution of diffraction anisotropy.

### Anisotropy is not linked to the function of the membrane protein

Following the separation initiated above, polytopic membrane proteins all-alpha, for which the largest amount of entries is available, were divided into sub-categories according to their function. The underlying idea is that transporters require large conformational changes to translocate their substrate across the membrane while some channels only require a side chain movement to allow the diffusion of ions through a pore. Five main subclasses were created: ATPases, Electron-transfer, Channels, Receptors and Transporters. Figure S6b unexpectedly yet unambiguously reveals that no statistical differences can be observed among these subclasses.

### Membrane proteins fully embedded or with extra-membranous domains display identical anisotropy

**Figure 4:**
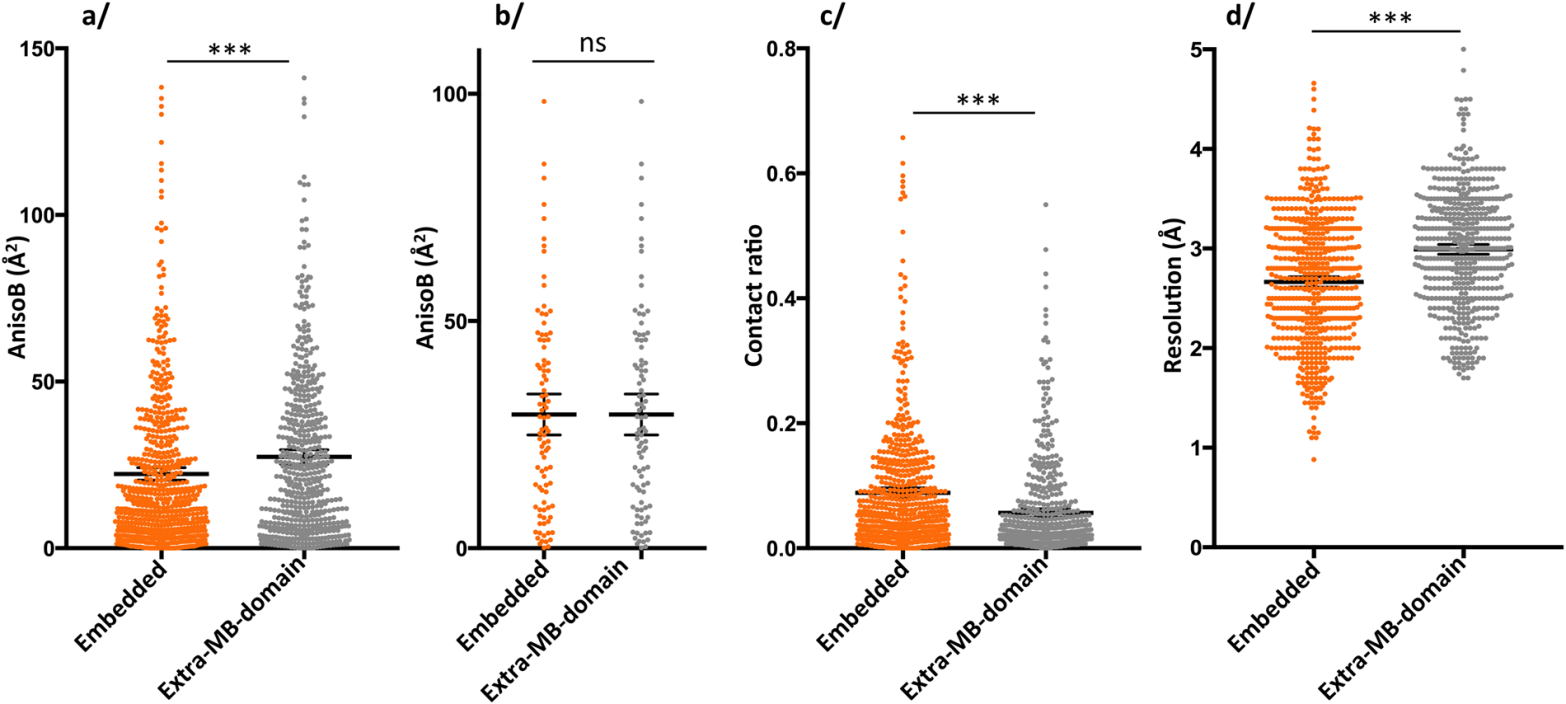
Comparaison membrane proteins fully embeded compared to those having extra-membranous-domains. (**a)** AnisoB: T-test with Welch correction two-tailed p-value = 0.0005; mean embedded = 22.3 Å^2^, mean extra-membranous-domains = 27.4 Å^2^; M-W two-tailed p-value = 0.0003. (**b**) AnisoB for structures between 2.8 and 3 Å resolution: T-test two-tailed p-value > 0.99, mean = 29.44 Å^2^; M-W two-tailed p-value > 0.99. (**c**) Crystal contacts: Student’s T-test with Welch correction: two-tailed p-value < 0.0001, mean embedded = 0.089, mean extra-MB-domain = 0.057; M-W two-tailed p-value < 0.0001. (**d**) Resolution: Student’s T-test with Welch correction: two-tailed p-value < 0.0001, mean embedded = 2.67 Å, mean extra-MB-domain = 2.99 Å; M-W two-tailed p-value < 0.0001.

Architecturally, membrane proteins can be found either fully embedded in the membrane with only small loops extruding, or having domains protruding outside of the membrane environment. We separated membrane proteins in these two categories irrespective of their function. For each PDB entry of our database, we rendered a molecular view using data from the ‘Orientation of Protein in the Membrane’ (OPM) server^17^. They were manually inspected to segregate proteins with respect to the presence of an extra-membranous domain. In this way, membrane proteins belonging to the alpha-helical or beta-barrel type of membrane insertion are mixed in the two categories. Note that protein fusions, such as T4 lysozyme typically used for GPCR, nanobody complexes, or complexes with other soluble partners are included in the category of “extra-membranous domains”. At first look, membrane proteins fully embedded in the membrane display less anisotropy than the ones having extra-membranous domains (Fig. 4a). The difference in mean is again small, like the one observed for proteins crystallized in detergent or LCP, here between 3.5 and 6.5 Å^2^, but the statistical significance is identical for both parametric and non-parametric statistics. The distribution of anisotropy is very broad for both categories, as observed in the other analysis conducted so far. An explanation for this observation comes from the fact that fully embedded membrane proteins crystals diffract to higher resolution than the ones with extra-membranous domains (Fig. 4d), and that the former have more crystal contacts than the later (Fig. 4c). Again, these two observations go completely against the ideas accepted in the field. When comparing structures obtained at similar resolution (Fig. 4b), it is apparent that the difference in anisotropy disappears to yield the conclusions that these two categories of membrane proteins are indistinguishable in regard to diffraction anisotropy.

### Diffraction anisotropy and presence of ligands

The presence of a ligand would tighten the structure, resulting in less overall flexibility, and potentially having an effect on diffraction anisotropy. Amongst membrane proteins, 169 do not present any ligand and 1246 entries have been crystallized with one or more ligands. For our study, we defined a ligand as any heteroatom seen in the structure without requiring it to be located in the active site or buried in a pocket of the protein. We chose this segregation as diffraction is a measure of the whole assembly, and the presence of a ligand even at the periphery would have an effect on local flexibility. For example, a detergent molecule visible in the electron density map is rigid enough to be present in the X-ray diffraction, which reflects some local rigidity, and influences the protein around it. A wide distribution of diffraction anisotropy is again observed for membrane proteins Apo or in the presence of ligands (Fig. S6c). There is a small statistical difference between the two categories indicating that the presence of ligands would decrease anisotropy, given by the T-test, but this finding is challenged by the distribution of medians and the Mann-Whitney test that is not statistically significant. We thus extracted all the proteins that have been crystallized several times in the Apo and liganded forms, with the criteria of having at least two entries in each category (Table S3). For all these 17 proteins, no statistical difference exists between the proteins in the Apo and Holo forms. The distributions of anisotropy are identical within each protein. This result shows that ligands have no influence on the diffraction anisotropy.

## Discussion

Diffraction anisotropy has complicated the structure solving process for many years. Surprisingly, it has seldom been thoroughly investigated to find the origin of the phenomenon. We chose to explore this field of research with a special emphasis on membrane proteins, because they carry the advantage of having an intrinsic orientation; the membrane insertion defines a plane to which protruding portions are perpendicular. They also offer the convenience of being not too populated in the PDB, which allows the knowledge of their structures compared to the multitude of soluble proteins.

First and foremost, our study reveals that soluble and membrane proteins do not behave identically in their X-ray diffraction patterns. For both populations, the diffraction anisotropy can be fitted with a Weibull distribution but with different scale and shape parameters. In itself, this observation is major. This difference reveals the fact that these two categories of proteins are intrinsically distinct by obeying distinct sets of rules revealed by X-ray diffraction. Instead of seeing soluble and membrane proteins together as proteins but populating different environments, this result rather brings forward the concept that they are distinct objects made of the same material, amino-acids. Besides, the Weibull distribution is notably used in failure analysis to determine the properties of brittle materials. The analogy with protein is tempting, where anisotropy would be a measure of protein deformability. We here show that membrane proteins display more anisotropy than soluble proteins, and are more populated towards high anisotropy at a given resolution (Figs 1 and 2). In parallel, it should be noted that many studies highlight the flexibility of membrane proteins^18–20^, as well as the importance of the membrane lateral pressure on the function of membrane proteins^21,22^. Diffraction anisotropy could therefore be a reporter of the intrinsic flexible nature of these proteins.

We provide the values of anisotropy corresponding to the 25^th^, 50^th^ 75^th^ and 95^th^ percentile for a given resolution. This tool will help investigators to define how severe is the anisotropy of a dataset, and allows to position it with respect to others already available. It should also be considered that resolution is a user-defined parameter and therefore holds intrinsic bias. It is understandable that crystallographers facing low-resolution data, for example 3.5 Å resolution in the best diffracting direction, will do their best to gather the extra fraction of Å to get the best out of their data and to improve their electron-density maps. They would also spend extra time to try to optimize their crystals so that they diffract better. The same effort is not necessarily put forward when crystals are diffracting well at once. This could lead to a “crystallographer-bias” reporting better resolution for their data at low resolution. This bias would be more represented for membrane proteins as soluble proteins diffract to better resolution (average 2.76 Å or 2.13 Å respectively). To lift the veil on this potential bias, data should be handled the same way, which is now achievable since intensities are being deposited and we will investigate this in downstream studies. It should be noted though that considering this bias, we would only expect a stronger difference in anisotropy for these two types of proteins.

From our analysis, it appears that the number of crystal contacts is definitely a parameter influencing anisotropy, following the accepted view that more contacts will give a more constrained structure with less diffraction anisotropy. There is however a wide spread of anisotropy for any given amount of crystal contacts that reveals other phenomena at play (Fig. S2). This is brought forward by the comparison of membrane proteins crystallized in detergents or LCP that yield similar anisotropy. This striking result is rich of teachings. Membrane proteins are purified in detergents that surround their hydrophobic region to prevent aggregation. Since these detergents form a bulky and dynamic corona around the protein, they tend to reduce the crystal contacts, leading to high anisotropy. However, when detergents are replaced by lipid-like molecules during the crystallogenesis process of insertion into a lipidic cubic phase, it results in a tighter crystal packing and more crystal contacts. Yet, no difference in anisotropy can be found, especially when comparing the same proteins crystallized with both methods. Besides, the type of packing is very different between the two techniques of crystallization. If it can be argued that type II crystallization for membrane proteins surrounded by detergents would result in directional crystal contacts (because detergents prevent contacts), it is clearly not the case for crystallization in LCP^23,24^, where contacts are found laterally *via* lipids and vertically to another layer of protein.

Following this discovery, the directionality of crystal contacts resulting in directional diffraction is also questioned by the comparison of membrane proteins fully embedded in the membrane compared to the ones having extra-membranous domains, having identical anisotropic distributions. This finding is opposite to the movement going on in the field of membrane proteins exemplified for the study of GPCRs, where nanobodies are generated and fusions with T4 lysozyme are created. While inserting extra domains or crystallizing in LCP are indisputably essentials in the toolkit to obtain a membrane protein crystal, we show that it will not result in a better diffracting crystal than the one obtained in detergent. A further thought originating from this is the belief that there is a need to manipulate membrane proteins to render them more prone to crystallizing (including nanobody complexes, thermostabilization, mutation, protein fusions, etc…). If the difference in anisotropy is already observable for stabilized versions of membrane proteins, the question is open to what the difference would be if only non-modified proteins would be analyzed, but the gap would only expand from the actual measurement strengthening our findings.

We could not see an influence of the type of membrane protein, or their function on the level of anisotropy, nor the presence of ligands that would rigidify the structure. All together, these results correct many false preconceptions in the field of crystallography.

These findings are to be put in the perspective of the wide distribution of anisotropy observed in Figs 2 and S1, with extreme cases for soluble or membrane proteins, and overlapping anisotropy. The reasons for the appearance of this physical phenomenon are probably multiple, requiring case-by-case investigation, with resolution and crystal contacts having a strong impact. Undoubtedly, there is stronger anisotropy for membrane proteins, and our analysis points to the fundamental difference between a membrane and a soluble protein, i.e. the hydrophobic region that permits the insertion into a biological membrane.

Decades of biochemistry have already shown the need for specific tools to handle membrane proteins, from their recombinant expression to purification and crystallization. The development of specific tools has been needed to manage the experimental handling of membrane proteins, explaining the lag of their structures compared to the soluble proteins. The need for thermostabilization of GPCRs is a prime example of intrinsic flexibility, which is notably achieved through mutations of residues within the membranous region. The present study brings to light the translation of protein flexibility into X-ray diffraction and the prominent appearance of diffraction anisotropy. Overall, all these data reveal that membrane proteins are completely different objects than soluble proteins and call for a change of view on the way they are described, together with the ever more prevalent need to develop specific tools to handle them, also in the field of crystallography.

## Materials and Methods

### PDB data mining and curation

The detailed explanation on how the data was retrieved and processed, along with the database generated are available in the accompanying manuscript^25^. Briefly, a local copy of the RCSB Protein Data Bank (PDB)^26^ was made including all the deposited structures in PDB formatted coordinate files as well as all the crystallographic structure factors in mmCIF format, as of February 24^th^, 2016. This local copy was processed using software from the CCP4 software suite version 7.0^26,27^ and in-house automated Linux script to calculate the anisotropic delta-B statistic (‘aniso_b’ value) for each entry using the latest version of Phaser^14^ (on amplitude and intensity data when available) and the procedure described on the UCLA anisotropy server^2^, and each entry was put in the context of data already available by the PDB (such as resolution, space group, solvent content, etc…). We also calculated the ratio of crystal contacts for each entry, the ratio of number of contacts between the asymmetric unit and symmetric counterparts divided by the number of atoms in the asymmetric unit. The entries were then divided between soluble and membrane proteins, and the later was subdivided in several sub-categories based on keywords and interrogation of the ‘Membrane proteins of known structures’ database (http://blanco.biomol.uci.edu/mpstruc/) leaded by S.H. White (University of California, Irvine). These subsets are: soluble proteins; membrane proteins; membrane proteins structures solved in detergents, lipidic cubic phase (extracted as described by M. Caffrey^23^) or bicelles; α-helical or β-barrel transmembrane proteins; monotopic membrane proteins; membrane ATPase, electron-transfer, channel, receptor and transporter proteins. Finally, two other subsets (embedded membrane proteins and proteins with extramembranous domains) were extracted based on spatial arrangements information from the ‘Orientations of Proteins in Membranes’ (OPM) database^17^. Subsequent statistical analyses and charts rendering were conducted using Excel 2013 (Microsoft Corporation) and PRISM 7 (GraphPad Software, Inc).

The curation of the data was based on the plot of anisotropy over the years (Fig. S6d) showing that anisotropy increases after 1990 to reach a plateau around 2002 for the bulk of the entries, with a few cases displaying very high anisotropy. This phenomenon can most likely be attributed to software improvements and to their capacity to better handle this phenomenon, therefore enabling users to tackle more severe diffraction anisotropy. Severe diffraction anisotropy may have been encountered just as frequently before 2002 as it is now, but such data sets may never have been published because crystallographers were unable to achieve acceptable structure refinement statistics without the aid of post-2002 software. Note that most membrane proteins structures were mainly obtained after 2005. They would also benefit from software improvements and display high anisotropy that would bias their distribution towards high anisotropy. In the follow-up analyzes of this article, we thus focused only on the structures obtained after 2005 in order to compare structures of similar difficulty levels and susceptible to have comparable anisotropic behavior. Also, in order to compare reasonably well-behaved structures, only data diffracting to less than or equal to 5 Å resolution were kept, and anisotropic delta-B values above 150 Å^2^ were rejected. In addition, all crystal contacts ratio over 1 were removed from the analysis. Thus, our final dataset consisted of 76,458 entries with 74,928 and 1,411 calculated anisotropic delta-B values on amplitudes (soluble and membrane proteins, respectively); and 23,125 and 487 values on intensities.

### Statistical analysis

#### General statistics

The large number of entries gives a good view of the distribution of anisotropy, and clearly reveals a non-normal distribution, negatively skewed as given by the Pearson’s moment coefficients of skewness. Nevertheless, the large number of data grants the use of parametric statistics such as Student’s T-test, used here with the Welch correction when samples have unequal variances and unequal sizes^28^. Similarly, Analysis of variances (ANOVA) was used to compare variances between groups, with care to keep equal sizes in the groups (cf. below). Non-parametric tests were nonetheless conducted in parallel, Mann-Whitney to test medians, and Kruskal-Wallis for analysis of variances.

#### Statistical analysis of types of membrane proteins

The amount of PDB entries between the categories are not even. In order to investigate the analysis of variance between these categories, the same amount of entries were compared in the ANOVA; for the categories with higher number of entries (Channels, Receptors, transporters compared with ATPases and Electron-transfer; ALPHA and BETA *vs* MONO), the entries were randomized and the same amount as the lowest category was extracted. ANOVA is indeed quite sensitive to unevenness of the samples to be tested, and resulted in false positive results when analyzing the whole dataset^29^. Reducing the dataset to the smallest amount of structures gave unambiguous negative results.

#### Statistical testing of membrane proteins crystallized in different types of methods

Note that membrane proteins crystallized *in-meso* from bicelles were not included in the study owing to their small representation (30 entries). This number is at the border defined by Student where the T-test is not sensitive to normality; therefore, the foreseen skewness of their distribution would have resulted in un-trustworthy results. Pair-wise comparison were thus conducted between membrane proteins crystallized in detergents *vs* LCP.

### Fit of anisotropic delta-B values over resolution

The anisotropic delta-B value associated with each entry was plotted against its resolution value (Fig. 2). The anisotropic values were divided in overlapping bins as a function of the resolution, each bin being 0.5 Å large and successive bins displaced of 0.1 Å along the resolution in the range 0–4 Å. The 25^th^, 50^th^, 75^th^ and 95^th^ percentile of the distribution of anisotropic values within each bin was calculated (filled dots in Fig. S5). The 97.3^th^ and 99^th^ percentiles were also calculated for the subset of soluble proteins. Finally, by considering the resolution within each bin constant and equal to its mean value, the dependency of the anisotropy on the resolution at each percentile threshold was described with a power law of the type *y* = *ax* ^*b*^ + *c*, where *y* is the anisotropy expressed in Å^2^ and *x* the resolution expressed in Å. The parameters *a*, *b* and *c* (the latter being the extrapolated anisotropy at zero Å resolution, it reflects the uncertainty in the fitted data) were obtained by a non-linear least square fit performed with the Curve Fitting Toolbox of Matlab_R2014b. The values of *a*, *b* and *c* at each percentile threshold can be found in Fig. S5.

## Electronic supplementary material

Supplemental information
