# Supplementary material for: X-ray diffraction reveals the intrinsic difference in the physical properties of membrane and soluble proteins
Source: Sci Rep. 2017 Dec 5;7:17013. doi: 10.1038/s41598-017-17216-1 (PMC5717104; doi:10.1038/s41598-017-17216-1)
Supplement: Supplementary file 1 — Supplemental information [file 41598_2017_17216_MOESM1_ESM.pdf]

Title: X-ray diffraction reveals the intrinsic difference in the physical properties of membrane and soluble proteins

Authors: Xavier Robert[1a], Josiane Kassis-Sahyoun[1a], Nicoletta Ceres[1a], Juliette Martin[a], Michael R. Sawaya[b], Randy J. Read[c], Patrice Gouet[a], Pierre Falson[a], and Vincent Chaptal[a*]

Legends supplemental-information

Fig. S1: comparison of anisotropy between soluble and membrane proteins. **a/** Calculation on amplitudes. Plot of all the entries on a scatter plot. Black bars show the average anisotropy of the sample and brackets display the 95% confidence interval of the population mean. Student's T-test with Welch correction (two-tailed p-value < 0.0001), and Mann-Whitney difference of medians (two-tailed p-value < 0.0001). **b/** Calculation on intensities. Plot of all the entries on a scatter plot. Black bars show the average anisotropy of the sample and brackets display the 95% confidence interval of the population mean. Student's T-test with Welch correction (two-tailed p-value < 0.0001), and Mann-Whitney difference of medians (two-tailed p-value < 0.0001).
The probability distribution function of these two types of proteins can be fitted by a Weibull distribution : y = a*b*x^(b-1)*exp(-a*x^b) ;

	On amplitudes (F)	On Intensities (I)
Soluble proteins	a = 0.15 ; b= 0.83	a = 0.05 ; b= 1.07
Membrane proteins	a = 0.05 ; b= 0.93	a = 0.02 ; b= 1.09

Fig. S2: comparison of crystal contacts between soluble and membrane proteins. **a/** Graph of anisotropy (calculated on amplitudes) as a function of crystal contact ratio for soluble (blue) and membrane proteins (red). Each dot represent an entry. The black dots represent the 95th percentile for each contact ratio bin, and has been fitted with a double exponential: y = a*exp(b*x)+c*exp(d*x)
Soluble proteins:
Coefficients (with 95% confidence bounds):
a = 20.39 (17.13, 23.65)
b = -0.2679 (-0.4945, -0.04127)
c = 44.4 (40.61, 48.19)
d = -10.21 (-12.1, -8.327)
Membrane proteins:
Coefficients (with 95% confidence bounds):
a = 79.22 (59.35, 99.1)
b = -3.639 (-4.653, -2.626)
c = 19.61 (-7.676, 46.89)
d = -55.22 (-249.9, 139.4)
b/ Scatter plot of soluble proteins in blue and membrane proteins in red, with black bars representing the mean of the sample and brackets displaying the 95% confidence interval of the population mean. Student's T-test with Welch correction (two-tailed p-value < 0.0001), and Mann-Whitney difference of medians (two-tailed p-value < 0.0001).

Fig. S3: comparison of solvent content between soluble and membrane proteins. **a/** Graph of anisotropy (calculated on amplitudes) as a function of solvent content for soluble (blue) and membrane proteins (red). Each dot represent an entry. The black dots represent the 95th percentile for each solvent content bin, and has been fitted with a Gaussian fit: y=a*exp(-((x-b)/c)^2);
Soluble proteins:
Coefficients (with 95% confidence bounds):
a = 64.06 (58.86, 69.27)
b = 85.56 (76.75, 94.37)
c = 44.24 (35.19, 53.28)
Membrane proteins:

Coefficients (with 95% confidence bounds):
a = 79.35 (71.89, 86.81)
b = 68.39 (66.56, 70.21)
c = 21.19 (18.44, 23.94)

b/ Scatter plot of soluble proteins in blue and membrane proteins in red, with black bars representing the mean of the sample and brackets displaying the 95% confidence interval of the population mean. Student's T-test with Welch correction (two-tailed p-value < 0.0001), and Mann-Whitney difference of medians (two-tailed p-value < 0.0001).

Fig. S4: comparison of resolution between soluble and membrane proteins. **a/** Graph of anisotropy (calculated on amplitudes) as a function of resolution for soluble (blue) and membrane proteins (red). Each dot represent an entry. **b/** Scatter plot of soluble proteins in blue and membrane proteins in red, with black bars representing the mean of the sample and brackets displaying the 95% confidence interval of the population mean. Student's T-test with Welch correction (two-tailed p-value < 0.0001), and Mann-Whitney difference of medians (two-tailed p-value < 0.0001).

Fig. S5: Power-law fits of anisotropy as a function of resolution. **a/** The four panels display the fits on amplitudes shown in Fig. 2A for comparison. The red curves corresponding to membrane proteins are always above the soluble proteins (blue). **b/** fits calculated on intensities as in Fig. 2B. Bellow are equations defining the percentile-fits of anisotropy (calculated on amplitudes or intensities) as a function of resolution.

	**Soluble proteins**		**Membrane proteins**	
	AnisoB(F) =	AnisoB(I) =	AnisoB(F) =	AnisoB(I) =
99th percentile	$6.68\times Reso^{2.22} + 4.28$	$15.3\times Reso^{1.71}$	-	-
95th percentile	$3.64\times Reso^{2.46} + 3.84$	$7.84\times Reso^{1.93}$	$5.07\times Reso^{2.32} + 1.93$	$8.63\times Reso^{2.11}$
75th percentile	$2.16\times Reso^{2.40} + 1.39$	$4.25\times Reso^{2.07}$	$2.95\times Reso^{2.34}$	$4.96\times Reso^{2.16}$
50th percentile	$0.94\times Reso^{2.55} + 1.01$	$2.4\times Reso^{2.13} + 0.08$	$1.57\times Reso^{2.44} + 0.11$	$2.89\times Reso^{2.28}$
25th percentile	$0.36\times Reso^{2.45} + 0.42$	$1.35\times Reso^{2.03}$	$1.32\times Reso^{1.78}$	$1.18\times Reso^{2.62}$

c/ Two types of membrane protein crystallization. Type I crystals: layers of membrane proteins stacked side by side, with hydrophobic surfaces providing crystals contacts, are stacked one on another; Type II crystals: detergent molecules shield hydrophobic regions of membrane proteins and crystal contacts involve only polar heads; The hydrophobic region of the protein is depicted here in green, and extra-membranous parts in orange. Fig. adapted from (24). **d/** Examples of proteins belonging to different classes of membrane proteins according to their insertion in the membrane. The proteins are depicted in blue cartoon representation, the membrane is shown in orange. All proteins are on the same scale. Alpha: Alpha-helical transmembrane proteins. GpA: Glycophorin A, *PDB code (2kpe);* LacY: Lactose permease, *PDB code (2y5y);* Atm1: *PDB code (4mrs);* A2A receptor: Adenosine receptor, *PDB code (3pwh);* Aqp0: Aquaporin 0, *PDB code (2z5x). Beta:* Beta-barrel transmembrane proteins: OprM: Outer, *PDB code (3d5k);* mVDAC-1: channel, *PDB code (3emn). Mono:* Monotopic membrane proteins: MoaB: Monoamine oxidase B, *PDB code (1s3e);* PGH2S1: Prostaglandine H2 synthase 1, *PDB code (1q4g).*

Fig. S6: a/ comparison of anisotropy as a function of insertion into the membrane. ANOVA of the three types of membrane proteins as defined by Stephen White's database. The three samples have been analyzed for samples of the same resolution range (2.5-3 Å) to avoid a bias due to the effect of resolution. No significant difference could be found using ANOVA or Kruskal-Wallis analysis. Average ALPHA = 22.75, BETA

= 24.01 and MONO = 17.27. **b/** Comparison of diffraction anisotropy between ATPases, Electron-transfer proteins, transporters, channels and receptors. The comparison has been conducted using the ANOVA with Tukey's post-Hoc analysis. The three samples have been analyzed with the same number of entries in each category, after randomization of the transporters, channels and receptors. No statistical differences have been observed. Means: ATPases = 22.06 Å^2, E-transfer = 22.84 Å^2, transporters = 25.51 Å^2, channels = 24.62 Å^2, receptors = 20.54 Å^2. **c/** Influence of ligands on diffraction anisotropy of membrane protein crystals. The horizontal bar represents the mean and the brackets the 95% confidence interval of the mean. Student's T-test with Welch correction (two-tailed p-value = 0.0058), and Mann-Whitney difference of medians (two-tailed p-value = 0.037). **d/** Anisotropy of macromolecule crystals over the years. For each year, the average anisotropy is shown as a red horizontal bars; note that the scale (right) has been magnified to show more clearly the values.

Figure S1

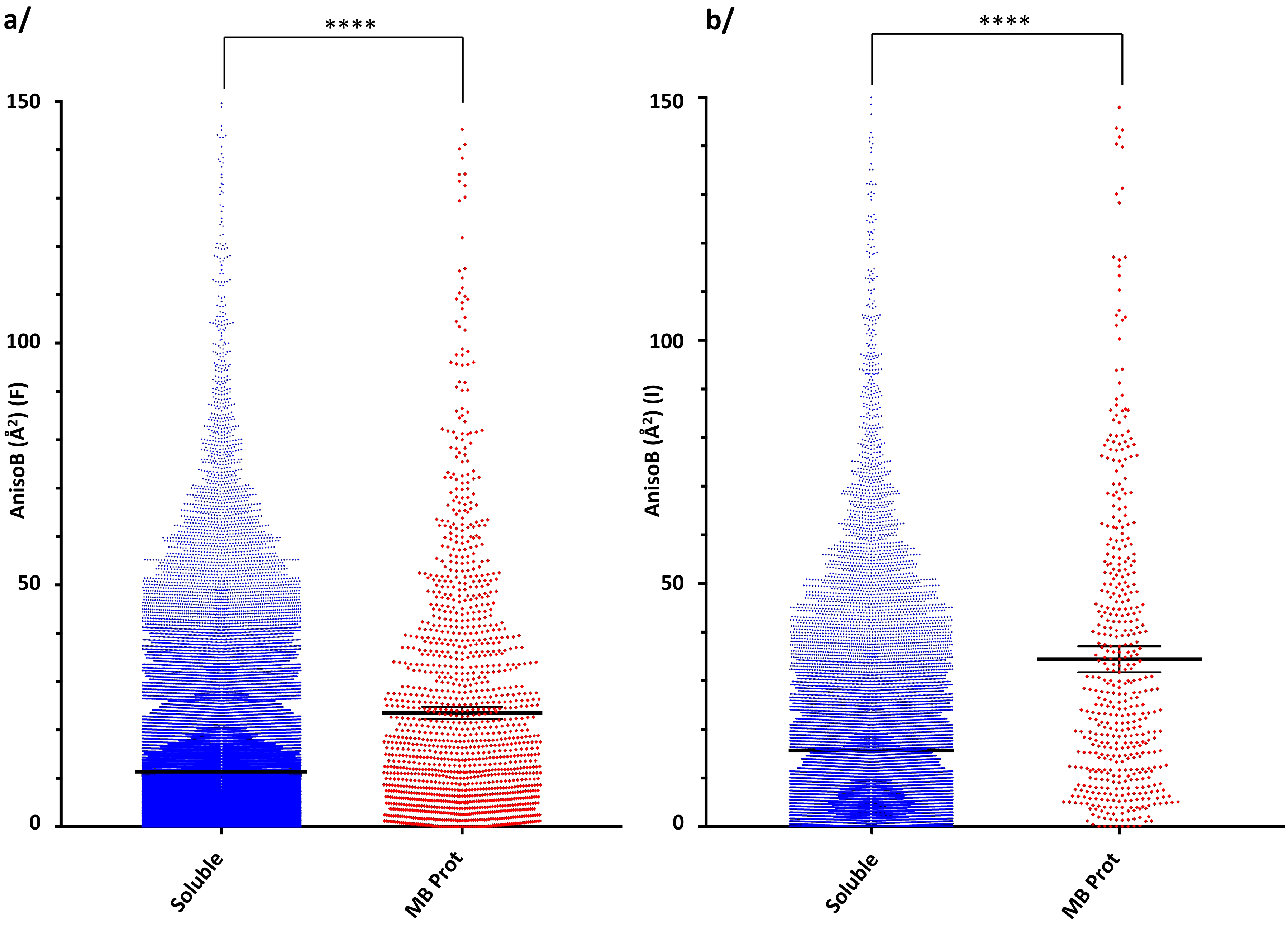

a/
****
150
100
50
0
AnisoB (Å²) (F)
Soluble
MB Prot
b/
****
150
100
50
0
AnisoB (Å²) (I)
Soluble
MB Prot

Figure S2

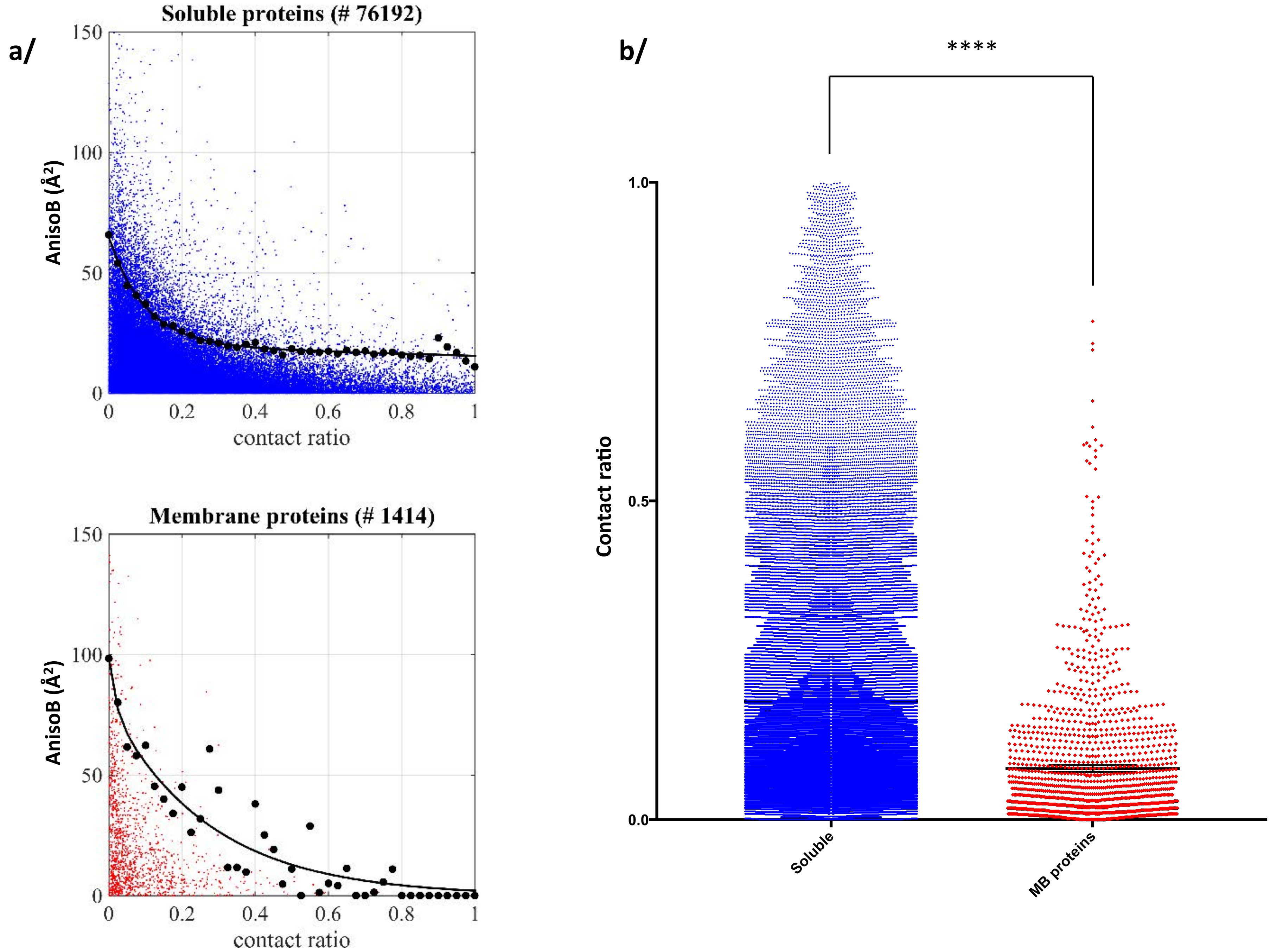
a/
Soluble proteins (# 76192)
AnisoB (Å²)
contact ratio
Membrane proteins (# 1414)
AnisoB (Å²)
contact ratio
b/
****
Contact ratio
Soluble
MB proteins

Figure S3

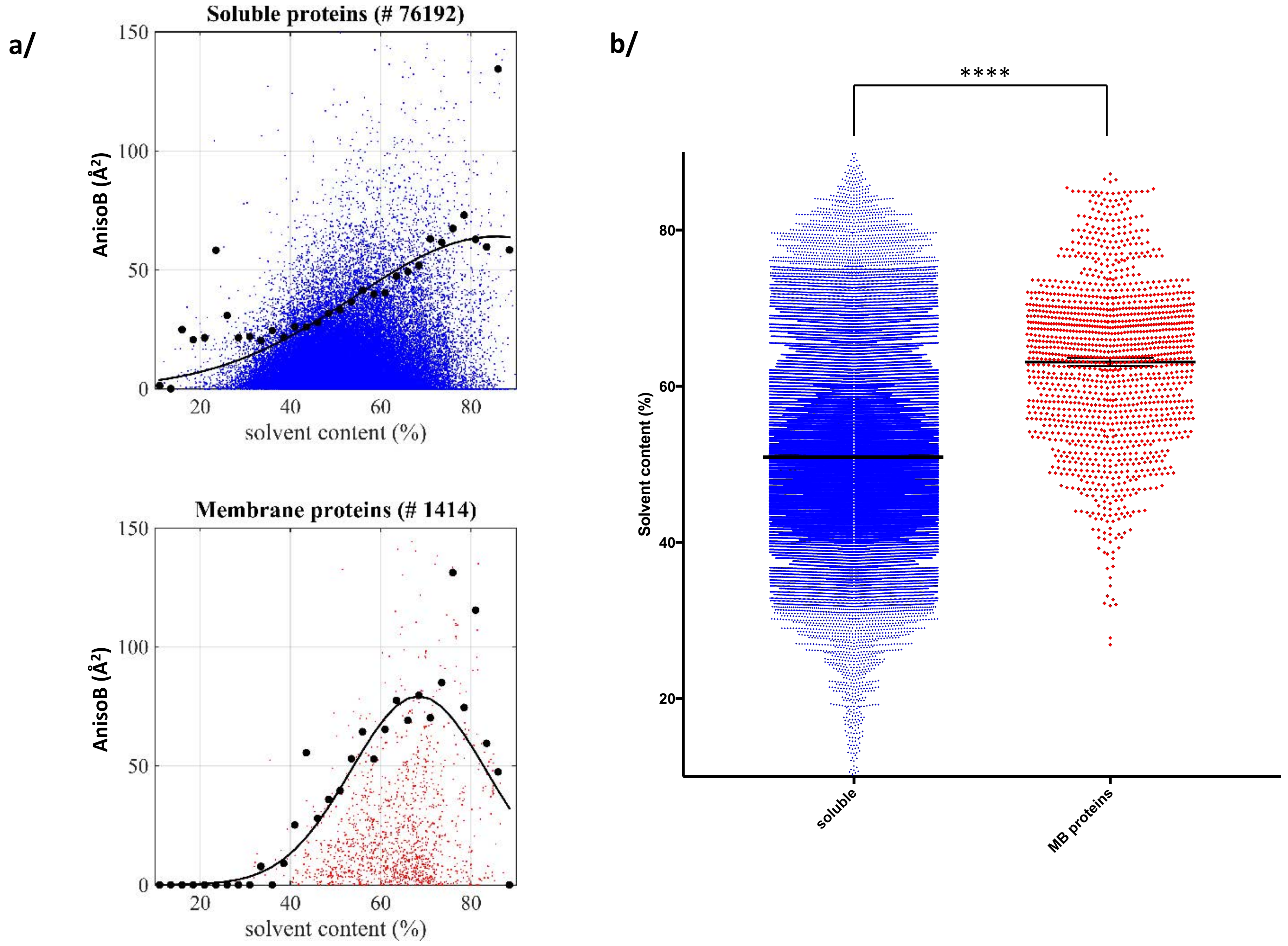

a/
Soluble proteins (# 76192)
AnisoB (Å²)
150
100
50
0
20
40
60
80
solvent content (%)
Membrane proteins (# 1414)
AnisoB (Å²)
150
100
50
0
20
40
60
80
solvent content (%)
b/
****
Solvent content (%)
80
60
40
20
soluble
MB proteins

Figure S4

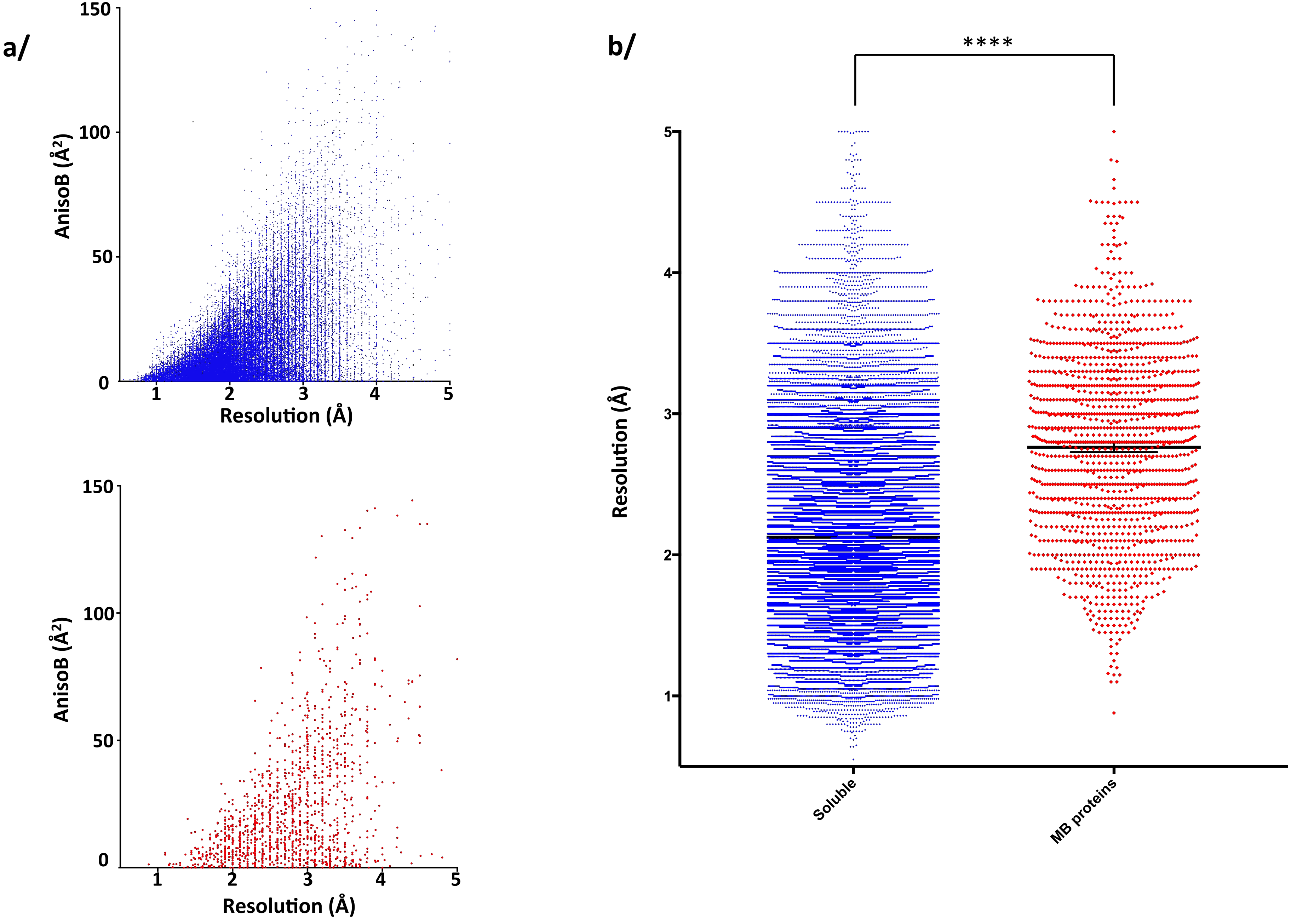
a/
AnisoB (Å²)
150
100
50
0
1
2
3
4
5
Resolution (Å)
b/
****
Resolution (Å)
5
4
3
2
1
Soluble
MB proteins

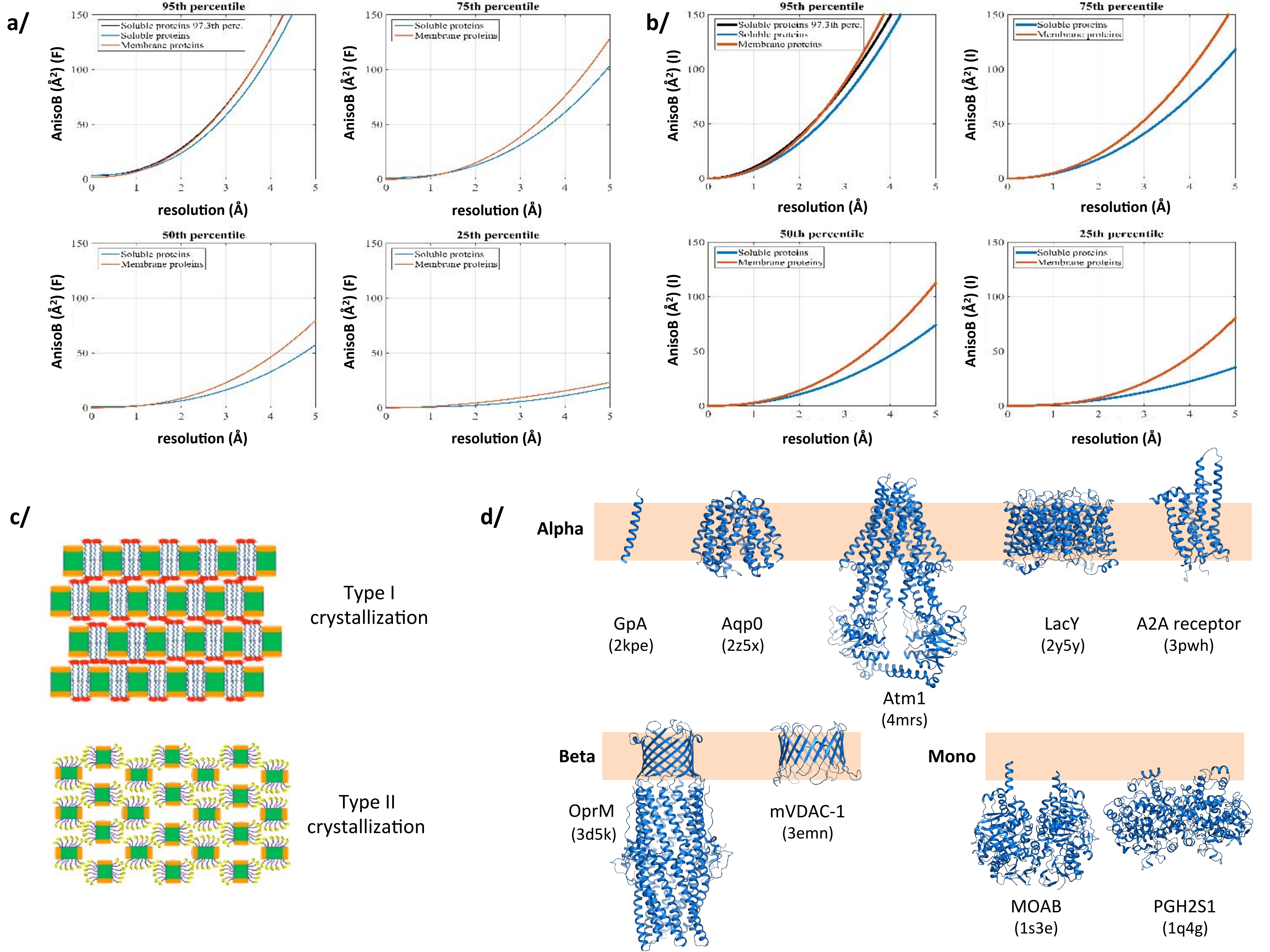
a/
95th percentile
75th percentile
50th percentile
25th percentile
Soluble proteins 97.3th perc.
Soluble proteins
Membrane proteins
AnisoB (Å²) (F)
resolution (Å)
b/
AnisoB (Å²) (I)
c/
Type I crystallization
Type II crystallization
d/
Alpha
GpA (2kpe)
Aqp0 (2z5x)
Atm1 (4mrs)
LacY (2y5y)
A2A receptor (3pwh)
Beta
OprM (3d5k)
mVDAC-1 (3emn)
Mono
MOAB (1s3e)
PGH2S1 (1q4g)

Fig. S6

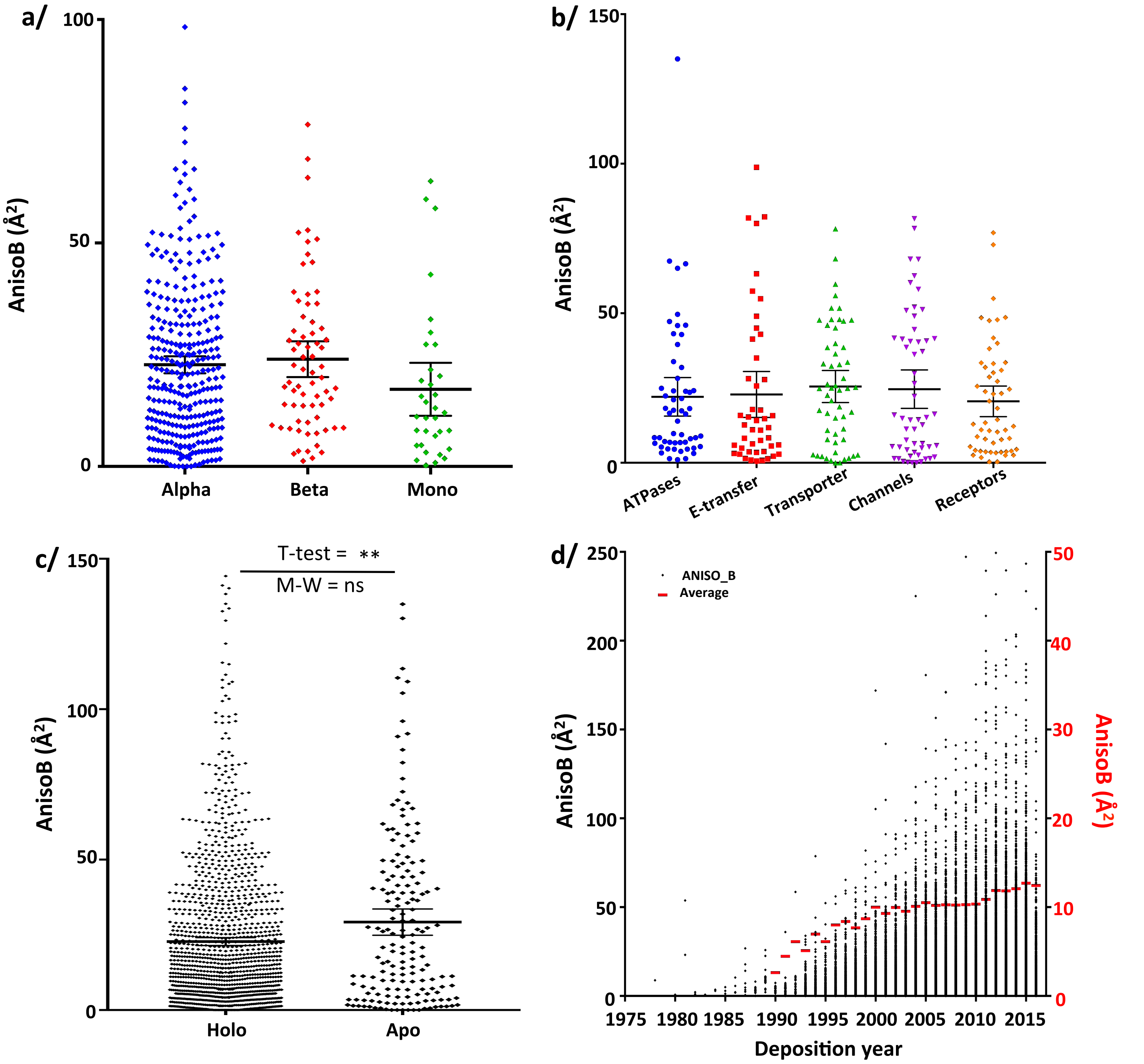
a/
AnisoB (Å²)
100
50
0
Alpha
Beta
Mono
b/
AnisoB (Å²)
150
100
50
0
ATPases
E-transfer
Transporter
Channels
Receptors
c/
AnisoB (Å²)
T-test = **
M-W = ns
150
100
50
0
Holo
Apo
d/
AnisoB (Å²)
ANISO_B
Average
250
200
150
100
50
0
50
40
30
20
10
0
1975
1980
1985
1990
1995
2000
2005
2010
2015
Deposition year

Table S1: General statistics

		Resolution (Å)		Ratio1		Solvent content (%)		Anisotropy (Å^2)	
	Number of entries	Mean	Median	Mean	Median	Mean	Median	Mean	Median
Soluble proteins	76192 structure factors	2.126 (2.122 – 2.13)	2.04	0.1855 (0.1844 – 0.1867)	0.136	51.11 (51.04 – 51.18)	50.16	10.72 (10.63 – 10.81)	6.39
	22985 intensites							*15.68 (15.57 – 15.79)*	*10.62*
Membrane proteins	1415 structure factors	2.763 (2.729 – 2.797)	2.8	0.08 (0.075 – 0.085)	0.042	63.11 (62.58 – 63.65)	64.22	23.53 (22.26 – 24.8)	15.15
	483 intensities							*34.43 (33.07 – 35.79)*	*25.83*
Membrane proteins in detergent	1241	2.79 (2.75 – 2.82)	2.8	0.078 (0.072 – 0.083)	0.037	64.19 (63.63 – 64.75)	65.35	24.1 (22.69 – 25.52)	15.1
Membrane proteins in LCP	173	2.61 (2.53 – 2.69)	2.6	0.096 (0.081 – 0.111)	0.069	55.64 (54.45 – 56.82)	55.83	19.53 (17.25 – 21.82)	15.46
ALPHA	1138	2.83 (2.79 – 2.87)	2.9	0.075 (0.069 – 0.08)	0.036	63.88 (63.27 – 64.48)	65.23	24.71 (25.68 – 26.02)	15.87
BETA	200	2.79 (2.41 – 2.57)	2.5	0.12 (0.099 – 0.13)	0.074	60.42 (59.19 – 61.67)	60.98	19.24 (16.82 – 21.66)	13.85
MONO	90	2.54 (2.63 – 2.54)	2.4	0.062 (0.052 – 0.072)	0.048	58.56 (56.92 – 60.21)	59.62	16.38 (12.89 – 19.87)	12.35
Transporters	272	2.99 (2.92 – 3.07)	3	0.049 (0.041 – 0.058)	0.025	65.04 (63.8 – 66.27)	67.3	31.46 (27.94 – 34.97)	24.36
Channels	225	2.78 (2.68 – 2.87)	2.94	0.11 (0.09 – 0.13)	0.038	66.25 (65.25 – 68.05)	67.87	24.65 (21.22 – 28.09)	14.6
Receptors	99	2.84 (2.74 – 2.93)	2.8	0.068 (0.055 – 0.081)	0.058	60.96 (59.01 – 62.9)	58.92	21.13 (17.4 – 24.86)	16.48
ATPases	54	2.99 (2.82 – 3.17)	2.95	0.042 (0.026 – 0.057)	0.024	63.43 (60.75 – 66.11)	64.86	22.06 (15.6 – 28.51)	16.45
Electron-transfer	46	2.87 (2.64 – 3.1)	2.93	0.032 (0.017 – 0.048)	0.019	67.47 (65.3 – 69.65)	68.16	22.84 (15.15 – 30.52)	12.19

The number in parenthesis represent the 95% confidence interval of the mean.

Table S2: List of proteins crystallized both in detergent and LCP.

	Crystallization in detergent		Crystallization in LCP	
Protein name	**PDB entry**	AnisoB (Å^2)	**PDB entry**	AnisoB (Å^2)
A2A adenosine receptor	**2YDV**	8.677	**3EML**	0.346
	**2YDO**	12.22	**4EIY**	2.476
	**4UHR**	11.333	**3QAK**	6.627
	**3VG9**	48.519		
	**4UG2**	10.018		
	**3VGA**	35.389		
AlgE alginate export protein	**4AZL**	17.505	**4AFK**	5.878
	**3RBH**	27.378	**4XNL**	27.483
	**4TQU**	29.299	**4XNK**	26.597
	**4TQV**	134.91	**4B61**	19.264
apo BtuB cobalamin transporter	**3M8B**	0.11	**2GUF**	25.8
	**3M8D**	0.13		
	**2GSK**	18.343		
	**2YSU**	40.021		
Bacterial homologue of ASBT (ASBTNM) with bound taurocholate	**3ZUX**	5.056	**4N7W**	14.94
	**3ZUY**	0	**4N7X**	20.815
Bacteriorhodopsin (BR)	**3T45**	6.384	**3NSB**	12.097
			**4FPD**	13.463
			**4X32**	3.924
			**3NS0**	12.746
			**4X31**	0.614
β2 adrenergic receptor	**3KJ6**	26.647	**2RH1**	20.474
	**2R4R**	39.25	**3D4S**	28.681
	**2R4S**	31.907	**3PDS**	41.713
			**4GBR**	71.171
			**3NY8**	30.945
			**3NY9**	28.964
			**3SN6**	1.636
			**3NYA**	33.284
			**3P0G**	72.903
Cytochrome C Oxidase aa3	**3HB3**	10.872	**2YEV**	14.112
	**3EHB**	11.017		
	**3X2Q**	14.736		
	**2GSM**	5.872		
	**3MK7**	3.132		
FraC eukaryotic pore-forming toxin from sea anemone	**4TSL**	4.517	**4TSY**	54.846
	**3VWI**	6.88		
	**3W9P**	9.453		
	**4TSN**	3.863		
	**4TSO**	17.297		
	**4TSQ**	0.03		

	**4TSP**<br>**3LIM**	5.41<br>8.863		
M2 proton channel (AM2)	**3C9J**<br>**3LBW**<br>**3BKD**	15.278<br>3.422<br>16.197	**4QK7**<br>**4QKC**	5.13<br>5.86
MgtE Mg^{2+} Transporter	**2YVY**<br>**2YVZ**<br>**2YVX**<br>**2ZY9**	15.728<br>62.254<br>27.04<br>57.848	**4U9N**<br>**4U9L**<br>**4WIB**	11.824<br>11.204<br>25.899
NarK nitrate/nitrite exchanger	**4JR9**<br>**4JRE**	7.759<br>9.593	**4U4W**<br>**4U4V**<br>**4U4T**	35.519<br>28.219<br>15.38
NTS1 neurotensin receptor in complex with neurotensin	**4BUO**<br>**3ZEV**<br>**4BV0**<br>**4BWB**	28.104<br>28.75<br>25.37<br>56.492	**4XES**<br>**4XEE**<br>**4GRV**	31.809<br>17.772<br>16.476
PepTSo Oligopeptide-proton symporter (POT family)	**4TPJ**<br>**4TPH**<br>**4TPG**<br>**2XUT**	1.667<br>2.599<br>2.262<br>8.682	**4UVM**	29.504
PepTSt Oligopeptide-proton symporter (POT family)	**4APS**	4.774	**4XNJ**<br>**4XNI**	17.906<br>14.703
Photosynthetic Reaction Center	**4N7L**<br>**4N7K**	3.077<br>5.987	**2J8C**<br>**2J8D**<br>**2WJM**<br>**2WJN**<br>**4CAS**	1.424<br>7.643<br>5.821<br>0.588<br>8.182
RPE65 visual cycle retinoid isomerase	**3FSN**<br>**4F30**<br>**4F3D**	13.538<br>21.878<br>7.892	**4F3A**<br>**4F2Z**	1.774<br>59.784
Stearoyl-coenzyme A desaturase (SCD1) in complex with substrate	**4ZYO**	25.788	**4YMK**	16.714
Translocator protein (TSPO)	**4RYJ**	56.148	**4UC1**<br>**4UC2**<br>**4UC3**<br>**4RYN**<br>**4RYQ**<br>**4RYM**<br>**4RYI**	8.056<br>19.401<br>12.382<br>10.555<br>6.76<br>52.38<br>23.376
Zn^{2+}-transporting four-helix bundle	**4P6J**<br>**4P6L**	0.461<br>0	**4P6K**	8.604

Table S3: Membrane proteins for which structures are available, Apo or in complex with a ligand, with more than two entries in each category.

	Apo		Holo	
Protein name	**PDB entry**	AnisoB (Å²)	**PDB entry**	AnisoB (Å²)
AcrB, bacterial multi-drug efflux transporter	**2HQC**<br>**2HQG**<br>**2HQF**<br>**2HQD**<br>**4CDI**<br>**3AOA**<br>**2DHH**	1.112<br>3.455<br>12.778<br>0.685<br>0.462<br>6.957<br>45.89	**2RDD**<br>**4DX5**<br>**4DX7**<br>**2W1B**<br>**3AOC**<br>**2HRT**<br>**2GIF**<br>**4U96**<br>**4DX6**<br>**2J8S**<br>**4U95**<br>**4U8Y**<br>**4U8V**<br>**4C48**<br>**2DRD**<br>**3AOD**<br>**2F1M**<br>**3W9H**<br>**3AOB**	33.109<br>8.284<br>20.154<br>19.04<br>10.979<br>5.384<br>14.027<br>7.434<br>9.1<br>24.021<br>10.926<br>11.016<br>14.985<br>0<br>29.286<br>6.593<br>21.537<br>6.745<br>12.665
BamB, component of the Bam β-barrel assembly machine	**3Q54**<br>**3PRW**<br>**2HY3**<br>**3Q7O**	14.964<br>3.439<br>3.377<br>0	**4XGA**<br>**3Q7N**<br>**3Q7M**<br>**3P1L**	7.826<br>14.372<br>3.302<br>7.98
β2 adrenergic receptor	**2R4R**<br>**2R4S**	39.25<br>31.907	**2RH1**<br>**4GBR**<br>**3PDS**<br>**3NY8**<br>**3NY9**<br>**3NYA**<br>**3D4S**<br>**3SN6**<br>**3P0G**<br>**3KJ6**	20.474<br>71.171<br>41.713<br>30.945<br>28.964<br>33.284<br>28.681<br>1.636<br>72.903<br>26.647
CusA, metal-ion efflux pump	**3NE5**<br>**3OOC**<br>**3K07**	17.835<br>86.491<br>16.984	**3OPO**<br>**3OW7**	108.412<br>114.948
CusC, heavy metal discharge outer membrane protein	**4K7K**<br>**4K34**	13.891<br>9.828	**4K7R**<br>**3PIK**	10.675<br>9.999
DgkA, diacylglycerol	**3ZE5**<br>**3ZE4**	11.112<br>40.456	**3ZE3**<br>**4UXZ**	5.192<br>4.733

kinase (DAGK)			**4UYO**<br>**4UXX**<br>**4UXW**	16.36<br>21.676<br>10.549
Fadl, long-chain fatty acid transporter	**2R88**<br>**2R89**	21.716<br>36.168	**3DWO**<br>**3PGS**<br>**3PGU**<br>**3PGR**<br>**3PF1**<br>**3DWN**<br>**2R4N**<br>**2R4O**<br>**2R4P**<br>**2R4L**	5.995<br>6.187<br>6.78<br>32.298<br>16.135<br>22.64<br>25.179<br>38.693<br>16.967<br>30.545
GlpG, rhomboid-family intramembrane protease	**2NRF**<br>**3ODJ**<br>**5F5D**	38.781<br>15.986<br>26.075	**4QO2**<br>**4QNZ**<br>**4QO0**<br>**3UBB**<br>**5F5K**<br>**5F5B**<br>**5F5J**<br>**3B44**<br>**3B45**<br>**2IC8**<br>**2XTU**<br>**2O7L**<br>**2XOV**<br>**2XOW**<br>**3TXT**<br>**2IRV**<br>**2XTV**<br>**2NR9**	11.812<br>7.445<br>11.409<br>18.036<br>35.322<br>19.882<br>24.204<br>5.611<br>5.662<br>13.493<br>4.349<br>17.439<br>4.385<br>18.331<br>44.921<br>2.07<br>3.989<br>5.519
H^+/Cl^- Exchange Transporter	**4FG6**<br>**2EXW**<br>**2EXY**	61.648<br>47.669<br>46.241	**2EZ0**<br>**4FTP**<br>**3NMO**<br>**4MQX**<br>**4ENE**	39.96<br>50.685<br>11.16<br>72.237<br>32.318
Heterodimeric ABC exporter TM287-TM288	**4Q7M**<br>**4Q7K**<br>**4Q4H**	5.56<br>13.359<br>2.125	**3QF4**<br>**4Q4A**<br>**4Q7L**	81.445<br>60.68<br>2.492
KcsA, Potassium channel H^+ gated	**3EFD**<br>**3EFF**<br>**3PJS**	1.716<br>14.359<br>27.158	**2ITD**<br>**3STL**<br>**2W0F**<br>**4UUJ**	5.428<br>14.732<br>7.076<br>2.231

			**2JK5**<br>**2BOC**<br>**2BOB**<br>**2NLJ**<br>**3GB7**<br>**3IGA**<br>**3OR6**<br>**3OR7**<br>**3HPL**<br>**3FB6**<br>**3F7Y**<br>**3F5W**<br>**3F7V**<br>**3FB5**<br>**3STZ**<br>**2ITC**<br>**3FB7**	15.103<br>18.26<br>11.207<br>23.302<br>0.095<br>11.934<br>16.416<br>8.807<br>18.782<br>19.991<br>11.341<br>10.761<br>10.964<br>17.709<br>19.731<br>14.718<br>0.813
NTS1, neurotensin receptor	**4BV0**<br>**4BWB**	25.37<br>56.492	**4XEE**<br>**4XES**<br>**4GRV**<br>**4BUO**<br>**3ZEV**	17.772<br>31.809<br>16.476<br>28.104<br>28.75
OmpF, outer membrane protein F	**2ZLD**<br>**3K1B**<br>**4D5U**<br>**3K19**	68.801<br>1.694<br>69.748<br>2.144	**3FYX**<br>**3O0E**<br>**2ZFG**<br>**3HW9**<br>**3NSG**<br>**3HWB**	9.673<br>62.567<br>11.059<br>7.401<br>76.49<br>50.273
ELIC, Prokaryotic pentameric ligand-gated ion channel	**2VL0**<br>**4TWH**	36.962<br>67.004	**4TWD**<br>**3RQW**<br>**4TWF**<br>**3RQU**<br>**3ZKR**	57.217<br>37.942<br>71.84<br>43.626<br>62.23
GLIC, Prokaryotic pentameric ligand-gated ion channel	**3EHZ**<br>**3EI0**<br>**4NPQ**	91.848<br>109.179<br>58.614	**4IL9**<br>**4ILA**<br>**4HFI**<br>**4ILB**<br>**4ILC**<br>**4IL4**<br>**3IGQ**<br>**4QH1**<br>**4QH5**<br>**3P4W**<br>**4F8H**<br>**3EAM**<br>**3P50**	33.678<br>61.213<br>36.234<br>35.295<br>53.295<br>81.197<br>6.434<br>85.773<br>58.982<br>44.481<br>68.071<br>10.055<br>50.889

			**4NPP**	50.097
			**2XQ9**	103.447
			**2XQ4**	129.462
			**2XQ3**	109.097
			**2XQ7**	111.443
			**2XQA**	104.485
			**2XQ8**	109.741
V1-ATPase	**3GQB**	1.378	**3W3A**	22.354
	**3A5D**	3.992	**3A5C**	4.63
			**3V6I**	5.432
			**3K5B**	47.236
